# Cerebrospinal Fluid Viral Load and Intrathecal Immune Activation in Individuals Infected with Different HIV-1 Genetic Subtypes

**DOI:** 10.1371/journal.pone.0001971

**Published:** 2008-04-16

**Authors:** Sahra Abdulle, Lars Hagberg, Bo Svennerholm, Dietmar Fuchs, Magnus Gisslén

**Affiliations:** 1 Department of Infectious Diseases, The Sahlgrenska Academy, Göteborg University, Göteborg, Sweden; 2 Department of Virology, The Sahlgrenska Academy, Göteborg University, Göteborg, Sweden; 3 Division of Biological Chemistry, Biocentre, Innsbruck Medical University & Ludwig Boltzmann Institute of AIDS Research, Innsbruck, Austria; University of Cape Town, South Africa

## Abstract

**Background:**

HIV-1 exhibits a high degree of genetic diversity and is presently divided into 3 distinct HIV-1 genetic groups designated major (M), non-M/non-O (N) and outlier (O). Group M, which currently comprises 9 subtypes (A-D, F-H, J and K), at least 34 circulating recombinant forms (CRFs) and several unique recombinant forms (URFs) is responsible for most of the HIV-1 epidemic. Most of the current knowledge of HIV-1 central nervous system (CNS) infection is based on subtype B. However, subtypes other than subtype B account for the majority of global HIV-1 infections. Therefore, we investigated whether subtypes have any influence on cerebrospinal fluid (CSF) markers of HIV-1 CNS infection.

**Methodology/Principal Findings:**

CSF HIV-1 RNA, CSF neopterin and CSF white blood cell (WBC) count were measured in patients infected with different HIV-1 subtypes. Using multivariate regression analysis, no differences in the CSF WBC count, neopterin and viral load were found between various HIV-1 subtypes.

**Conclusions:**

We did not find any subtype-dependent differences in the markers evaluated in this study.

## Introduction

The genetic diversity of HIV-1 and the biological properties of the different subtypes might be important factors in the pathogenesis, transmissibility and clinical progression of the infection[1]. Some studies have reported that HIV-1 subtype has no significant impact on disease progression [2]–[5], while others have provided evidence that differences might exist [6]–[8]. For instance subtype D has been suggested to have greater virulence than subtype A [9], [10]

HIV-1 infection is characterized by a spectrum of neurological complications throughout its course, occurring either as a direct consequence of the HIV-1 infection itself or due to opportunistic infections and/or tumours [11]–[13]. AIDS Dementia Complex (ADC), the most severe manifestation of HIV-1 central nervous system (CNS) infection, developed in approximately 20 % of patients with advanced HIV-1 infection before the widespread use of antiretroviral treatment[14]. ADC is considered the result of a complex interplay between the effects of viral replication and immune activation in the brain, which ultimately lead to neuronal injury and death [15]–[18].

Cerebrospinal fluid (CSF) HIV-1 RNA and neopterin are two of the most extensively studied surrogate markers of HIV-1 CNS infection. HIV-1 RNA can be detected in the CSF of virtually all untreated patients irrespective of disease stage [19]. Neopterin is a marker of cell-mediated immune activation produced by cells of the macrophage lineage upon stimulation by interferon-γ [20]. Increased CSF neopterin concentrations are found in HIV-1-infected patients with AIDS, ADC [21] or with opportunistic CNS infections, but moderately elevated levels are also frequently found in asymptomatic HIV-1-infected individuals [22].

There is very sparse information available on the role of different subtypes in HIV-1 infection of the CNS. Even though the majority of HIV-1 infections in the world are caused by other subtypes, our knowledge of HIV-1 related neurological disease is based mainly on subtype B. Moreover, the extent of HIV-1 induced neurocognitive impairment in regions where HIV-1 is highly prevalent remains largely unknown, although some recent studies have begun to address this issue[23]–[26]. These studies have shown varying results[27]. For instance, an Ugandan study showed an ADC prevalence of 31% in HIV-1 infected patients [25], while a blinded study of HIV-1 positive and negative Ethiopian subjects found no significant differences between the two groups[23]. Since Ugandan patients are predominantly infected with subtype D and Ethiopians with subtype C, this finding dictates further investigation into the role of subtype in HIV-1 CNS infection [27]. Subtype differences in co-receptor usage, disease progression rate as well viral diversity could predispose for differences in the propensity for neurological disease[24] .

The aim of this study was to determine whether subtype has any influence on CSF markers of infectious activity.

## Materials and Methods

### Participants

Archived data from 128 neurologically asymptomatic HIV-1 infected patients who underwent lumbar punctures in a prospective research cohort at the Department of Infectious Diseases, Sahlgrenska University Hospital, Göteborg, Sweden were included. The CSF was handled, analysed and stored immediately after collection according to protocol. Patients were specifically queried regarding neurological problems and clinically examined. The patients were either treatment naïve (n = 124) or had been off treatment for at least 6 months (n = 4). Demographics and clinical characteristics of the study participants are presented in Table 1.

**Table 1:** Demographic and clinical characteristics of the study patients.

	Genetic subtype					
	B	C	CRF01_AE	CRF02_AG	A	D
**Number of patients**	54 (42%)	34 (27%)	16 (12%)	9 (7%)	9 (7%)	6 (5%)
**Sex**						
Male	48	17	11	6	8	3
Female	6	17	5	3	1	3
**Origin**						
European	46	8	7	1	2	0
African	1	26	3	7	7	5
Asian	3	0	6	1	0	0
South American	4	0	0	0	0	1
**Transmission route**						
Blood products	1	2	1	0	0	0
Heterosexual	22	31	13	8	8	6
Homosexual	23	0	0	0	0	0
IVDU	8	1	1	0	1	0
Unknown	0	0	1	1	0	0
**Age (median, range) years**	37 (23–68)	33 (18–59)	37 (17–58)	30 (23–51)	34 (26–67)	36 (23–49)
**CDC stage**						
A	32	28	12	7	5	5
B	5	3	2	0	2	0
C	17	3	2	2	2	1

CDC–Centers for Disease Control and Prevention; IVDU–Intravenous drug use

### Laboratory methods

HIV-1 RNA was quantified in cell-free CSF and plasma using the Roche Amplicor Monitor assay (version 1.0 and 1.5, Hoffman La-Roche, Basel, Switzerland). Neopterin was measured in CSF and serum using a commercially available radio-immunoassay (Henningtest Neopterin, BRAHMS, Berlin, Germany). The normal reference value for neopterin was ≤5.8 nmol/L in CSF and ≤8.8 nmol/l in serum [28]. Routine assessments also included CSF white blood cell (WBC) counts and peripheral blood CD4 cell count determinations.

Subtypes were identified by the Stanford HIV Reverse Transcriptase (RT) and Protease (PR) Sequence Database (http://hivdb.stanford.edu) [29] using consensus RT and PR sequences obtained through an in-house method. Plasma HIV-1 RNA was extracted using the Magnapure Total Nucleic Acid isolation kit (Roche) according to the manufacturer’s protocol. The outer primers RT1 (5′-CTGTACCAGTAAAATTAAA-GCCA-3′), RT2 (5′-TATGTCATTGACAGTCCAGCT -3′), PR1 (5′- CCGATAGACAAGGAACT -3′) and PR2 (5′- TCTACTAATGCTTTTATTTTT -3′) were used in the first round polymerase chain reaction (PCR). The reaction mixture contained 8 units RNasin® (Promega), 4 units AMV (Promega), 200 mM of each dNTP (Roche), 1 unit AmpliTAQ DNA polymerase (ABI), anti-TAQ as recommended by the manufacturer (Clontech), 200 nM of the each of the outer primers, 60 mM KCl, and 2.0 mM MgCl2 in 20 mM Tris-HCl buffer. The amplification program in the first PCR cycle was 60 min at 43°C then 2 min 30s at 94°C followed by 40 cycles with settings 20 s at 94°C, 20 s at 50°C, and 60 s at 72°C. The final cycle was 5 min at 72°C and a final incubation at 8°C.

The next step was a second (nested) PCR with a mixture consisting of 5 µl of the first PCR product and the same reagents as in the first PCR excluding RNasin® and AMV, and the inner primers RT3 (5′- ATGGCCATTGACAGAAGAAA3′), RT4 (5′- AGGCTGTACTGTCCATTTAT-3′), PR 3 (5′- ACTTCCCTCAGATCACTCTT-3′) and PR 4 (5′- TTCCTGGCTTTAATTTTACT- 3′). The amplification steps in the nested PCR were the same as in the first PCR except for the omission of the first step of 60s at 43°C. All PCR reactions were carried out in GenAmp® PCR system 9600 (ABI).

The nested PCR product was separated by agarose gel electrophoresis of 15 µl of the master mix on 1.8 % agarose, containing 0.25 µg ethidium bromide per ml, and visualized by UV illumination.

The PCR product was purified using QIAquick PCR Purification Kit (Qiagen) prior to cycle sequencing with Big Dye Terminator Ready Reaction Kit version 1.1 (ABI Prism).

Nucleotide sequences were analyzed on the ABI Prism 3100 Genetic Analyzer sequencer and aligned with Sequence Navigator software.

### Statistical analysis

Plasma and CSF HIV-1 RNA were log_10_ transformed before statistical analysis. Because of the skewed distributions of several of the study variables, median and interquartile range (IQR; 25th -75th percentile) were used for descriptive statistics. Mann-Whitney’s (two independent samples) and Kruskall Wallis (several independent samples) tests were used for comparisons. Spearman’s rank correlation coefficient was used for evaluation of correlations. Linear multiple regression was performed on the CSF markers to evaluate the effect of different subtypes. CSF neopterin was log transformed for the multivariate analysis. Four outliers were removed from the CSF WBC count before the multivariate analysis. The removal of outliers was the best way to transform the CSF WBC count data and was done by a statistician. A P-value of <0.05 was considered significant.

## Results

**Figure 1:**
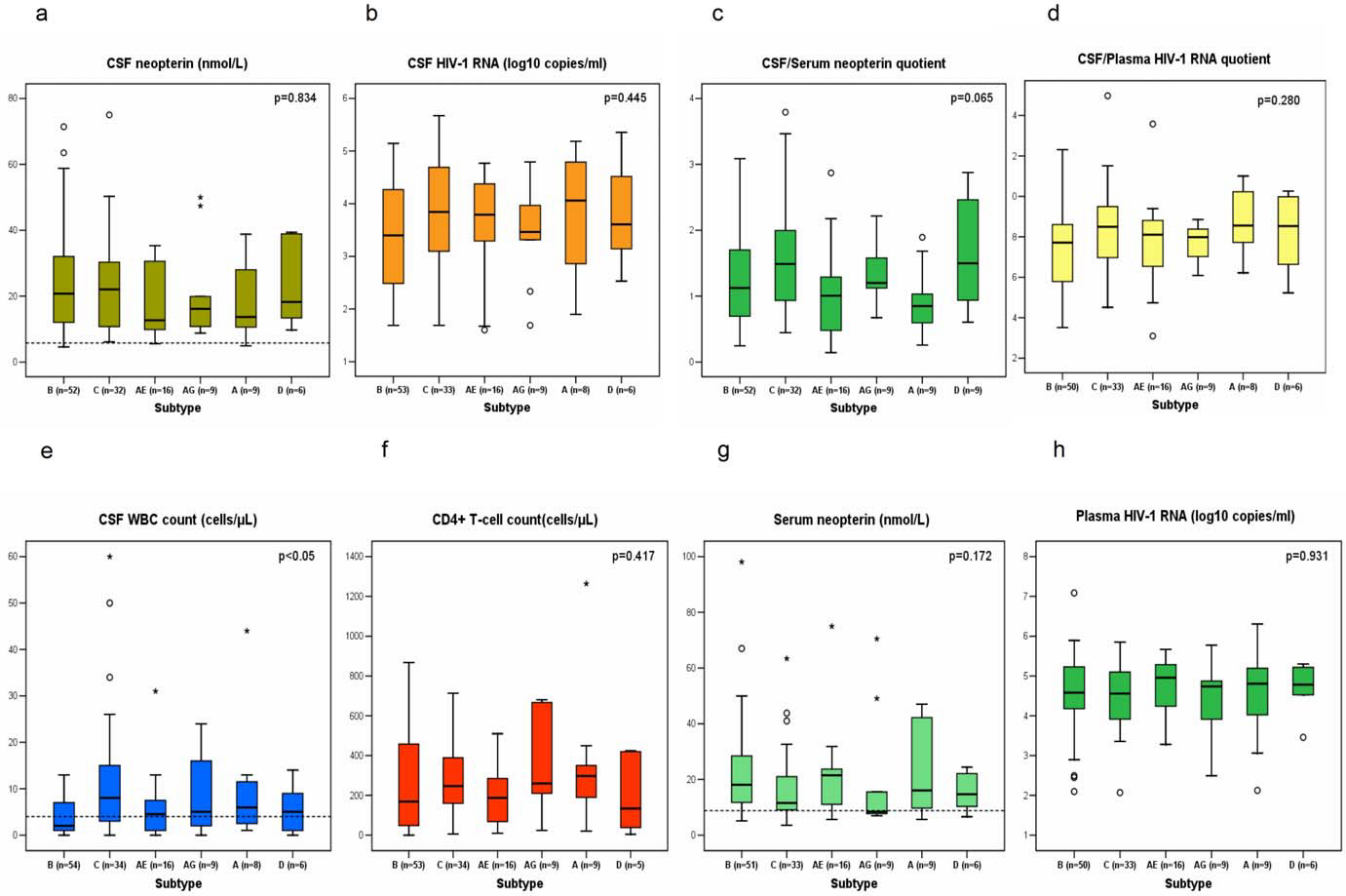
Boxplots of the cerebrospinal fluid (CSF) and plasma/serum levels of HIV-1 RNA and neopterin, the CSF/P-RNA and CSF/S-neopterin quotients, the CD4+ T- lymphocyte and the CSF white blood cell (WBC) counts according to subtype. The number of available samples is shown by n. The P-values on the top right of each panel are calculated using Kruskall-Wallis test. The bar inside the box shows the median values, the bottom and top hinges of the box represent the 25th and 75th percentile. The dotted lines represent the normal reference values. Outliers are depicted by o, extremes by *. Circulating recombinant forms CRF01_AE and CRF02_AG are designated as AE and AG.

Subtype was successfully assigned in both the RT and protease sequences in all but six samples that could be identified only by protease (2B, 2A, and 1CRF01_AE) or by RT (1CRF01_AE) sequence analysis. Figure 1 shows the results of the descriptive statistics for HIV-1 RNA and neopterin in CSF and plasma/serum, the CSF WBC count, the CSF/blood HIV-1 RNA and neopterin quotients and the CD4 cell count. All but four patients had elevated CSF neopterin concentrations. The median CSF neopterin levels (Panel A) were highest in patients with subtypes B and C but this difference did not reach statistical significance. Seven out of the 128 patients (5%) had CSF HIV-1 RNA below the detection limit of 50 copies/ml. Panel B and D show the CSF HIV-1 RNA and the CSF/plasma HIV-1 RNA quotients with median values that were in the same range in all the subtypes. The median CSF/serum neopterin quotients in panel C were higher in subtypes C and D although this difference was not significant. Most of the patients with subtype C and half of those with subtype D had significantly higher neopterin levels in CSF than in serum, which would explain the higher CSF/serum neopterin quotients. This was independent of disease stage.

A significant subtype dependent difference was found in the CSF WBC count (p<0.05) in the Kruskall Wallis test (Panel E). A statistically significant difference in the CSF WBC count was found between subtype B and subtype C (p<0.05) when comparing individual subtypes. However, subtype did not prove to be a significant independent predictor for any of the CSF markers in the multiple regression analysis when all the other CSF markers, CD4+ T- cell count, plasma viral load, serum neopterin, patient origin, age and sex along with subtype were included as independent variables (data not shown). The CD4 cell count, CSF and plasma HIV-1 RNA were significant independent predictors of CSF WBC count. Patients with subtype D had the lowest CD4 cell counts (Panel F) while those carrying the subtype CRF01_AE had the highest serum neopterin concentrations (Panel G). The median Plasma HIV-1 RNA levels (Panel H) were in the same range throughout the subtypes. A subtype-independent analysis showed a significant correlation between CSF HIV-1 RNA and CSF neopterin (r_s_ = 0.45, p<0.0001).

## Discussion

We did not find any significant subtype-specific differences in the neuromarkers investigated in this study. CSF HIV-1 RNA and neopterin are two of the most studied markers of HIV-1 CNS infection. These markers reflect processes implicated in the pathogenesis of HIV-1 infection of the CNS, which makes them appropriate to study. Although the number of subjects studied was relatively low, especially for subtypes other than B and C, there are no larger CSF studies published on HIV-1 subtypes and CSF markers of infectious activity. The number of patients without antiretroviral treatment included would be sufficient to detect any larger differences between subtypes. Minor differences are of course not possible to detect.

We found a statistically significant difference in the CSF WBC count between subtypes B and C in the univariate analysis where subjects with subtype C had more CSF cells than those with subtype B. This result could not be verified in the multivariate analysis when all other independent variables were taken into account. Subtype B included more patients with advanced disease compared to subtype C, which probably explains this difference.

A slightly increased CSF WBC count that wanes in advanced systemic infection is a frequent finding in HIV-1 infected patients with or without symptoms [30], [31]. In a subtype-independent analysis, patients with CD4 <50 had significantly less CSF pleocytosis (data not shown) than those with more preserved CD4 cell counts, which is in agreement with previous studies. The CSF pleocytosis was correlated to the CSF viral load and inversely correlated to the plasma viral load, which also is in agreement with previous findings [30], [31].

A limitation in the study is that no patients with ADC were included, mirroring the fact, that ADC is nowadays uncommon in areas with access to antiretroviral treatment. Another limitation is that disease duration was unknown in a majority of cases. A longitudinal prospective study design would have been preferable to identify differences. These days, studies of this kind are not possible to conduct due to treatment intervention.

There were more patients who had suffered an AIDS defining illness in the subtype B group compared to the other groups which in its turn is explained by the change in the epidemiology of HIV-1 infection in Sweden and most of Europe: in the Pre-HAART era the majority of patients were of European origin, carrying subtype B. In the HAART-era, new infections are mostly diagnosed in individuals who acquired their infection in other parts of the world where other subtypes are predominant. Differences in origin and gender between subjects with different subtypes were taken into account in the multivariate analysis.

If subtype has little impact on susceptibility to CNS HIV-1 infection, which factors are then important? Host factors, coexisting morbidities and co-infections in combination with viral virulence factors might be more important than subtype. As yet, the role of these factors has been very little studied. Why some patients suffer from high CSF viral loads and/or signs of intrathecal immune activation while others exhibit only minor signs of CNS infectious activity remains unknown.

### Conclusions

We did not find any differences in CSF biomarkers of CNS infectious activity between subtypes in neurologically asymptomatic, untreated HIV-1 infected individuals.
